# Thermally Insulating Polyimide Aerogel Skeletons Constructed by Hybridizing the Al–O Network Phase

**DOI:** 10.3390/gels11110929

**Published:** 2025-11-19

**Authors:** Mingkang Wang, Shuai Yu, Jing Wang, Xin Zhao, Changpeng Yang, Ran Wei, Chun Liu, Sizhao Zhang

**Affiliations:** 1 Thermal Control Technology Laboratory of Aircraft in Space Environment, Polymer Aerogels Research Center, Jiangxi University of Science and Technology, Nanchang 330013, China; 2 National Key Laboratory of Spacecraft Thermal Control, Beijing Institute of Spacecraft System Engineering, Beijing 100094, China

**Keywords:** polyimide aerogels, thermal insulation, dimensional stability, Al–O network phase

## Abstract

Polyimide aerogels have garnered considerable attention due to their high-performance combination of a lightweight nature, low thermal conductivity, and high mechanical strength, which renders them ideal candidates for thermal insulators in aerospace. However, the inherent conflict in achieving multifunctional (dimensional stability and mechanical stiffness at high temperatures, and highly efficient thermal insulation) integration presents a great challenge. Here, we present an aerogel skeleton construction strategy based on hybridizing an Al–O network phase to create multifunctional polyimide aerogels. The resulting aerogels partially constructed by Al–O network phase exhibit an outstanding resistance to high temperatures (shrinkage of 0.56% after experiencing 200 °C for 2400 s), which is markedly superior to that of conventional polyimide aerogels. The excellent insulation capability of the aerogel is reflected in its low thermal conductivity (0.0214 W m^−1^ K^−1^) and its ability to maintain a cold-side temperature of just 59.7 °C under a 150 °C heat source. Furthermore, remarkable enhancements in mechanical properties are found at high-temperature conditions, providing evidence for the compressive stresses of 0.329 and 0.394 MPa under 3% strain at the respective temperatures of 200 and 250 °C, showing a clear trend of enhanced compressive stress with rising temperature. These advancements in high-temperature stability and mechanical properties substantially broaden the scope for their potential applications in aerospace thermal protection systems.

## 1. Introduction

When an aircraft flies at high speeds, its friction with the air generates substantial aerodynamic heating on its surface [1,2]. This aerodynamic heating presents a serious threat to the safety of the sensitive internal equipment within the aircraft [3,4,5]. Therefore, utilizing efficient thermal protection materials is critical. In recent years, organic polymer aerogels, a class of materials first introduced in the 1990s, have developed rapidly and have attracted significant attention for such applications [6]. These materials are typically characterized by their combination of a lightweight nature, excellent thermal insulation properties, and high toughness [7]. Examples include a wide variety of polymer systems, such as phenolic resin aerogels [8], polyurethane aerogels [9], and cellulose aerogels [10].

Among these, polyimide (PI) aerogels, first documented by Leventis et al., stand out due to their exceptional overall performance [11,12]. During high-speed flight, the aerodynamic thermal effects experienced by different parts of the aircraft vary significantly, necessitating the application of tailored thermal protection methods and materials [2,13]. In particular, thermal protection systems are required for the downwind surfaces of the aircraft in the low-to-medium-temperature range (from 200 to 500 °C) to minimize thermal redundancy [14]. In these regions, the use of low-density, high-temperature-resistant, and efficient organic thermal insulation materials can substantially reduce the weight of the thermal protection system while addressing the issues of powdering and scaling-off commonly associated with silica aerogel composites [15,16].

Within this context, polyimide aerogels have gained significant attention due to their inherent exceptional thermal stability and mechanical properties [17]. Recent advances have enabled the successful fabrication of polyimide aerogels through the crosslinking of amino- or anhydride-based polyimide wet gels, followed by supercritical CO_2_ drying or freeze-drying processes [18,19]. This class of aerogel exhibits outstanding high-temperature resistance, superior mechanical properties, and excellent thermal stability, making it highly suitable for a wide range of applications in industries such as electronics, energy, and aerospace [20,21,22]. For example, in aerospace, they have been utilized as thermal insulation materials for batteries and electronic devices in Mars rovers [23].

However, when polyimide is formed into its nanoporous aerogel via the sol–gel process, its often inadequate high-temperature resistance leads to severe thermal shrinkage and structural collapse at temperatures above 200 °C [24]. Moreover, Wang et al. [25] found that conventional polyimide aerogels exhibit a significant decrease in compressive stress as temperature increases. At a strain of 3%, the compressive stress values of these polyimide aerogels at −50, 100, and 200 °C were 0.08, 0.06, and 0.04 MPa, respectively, showing a notable decline in the effectiveness of their mechanical properties at higher temperatures. Furthermore, they reported a linear shrinkage of 5.74% in the width direction, further underscoring the dimensional instability of the aerogel at elevated temperatures. These findings highlight a critical limitation of traditional polyimide aerogels for high-temperature applications.

To overcome these challenges, numerous researchers have endeavored to enhance the properties of polyimide aerogels by incorporating various crosslinking agents. Crosslinkers increase the degree of crosslinking between polyimide chains, thereby improving the structural stability and high-temperature resistance of aerogels. Common crosslinkers include 1,3,5-triaminophenoxybenzene (TAB), octyl-(aminophenyl)-trisilane (OAPS), 1,3,5-benzenetricarbonyltrichloride (BTC), 1,3,5-tri (aminophenyl)benzene (TAPB), and tris (4-aminophenylamine) (TAPA) [26,27,28,29,30]. These crosslinkers enhance the structural stability of polyimide aerogels by introducing additional chemical bonds between polyimide chains, thereby improving their high-temperature and compressive strength. For example, Xie et al. [31] proposed a dual-crosslinking strategy by using trisocyanates in combination with a polyimide matrix to successfully synthesize polyimide aerogels with enhanced mechanical strength and excellent thermal insulation properties. These studies suggest that the selection of appropriate crosslinker types and crosslinking densities plays a crucial role in enhancing the high-temperature performance, mechanical strength, and other physicochemical properties of polyimide aerogels. For instance, Simón-Herrero et al. [32] investigated the synthesis and characterization of linear and crosslinked polyimide aerogels, finding that crosslinked aerogels exhibited stronger thermal stability and mechanical strength. Moreover, Zhang et al. [33] studied the impact of low-cost polymer crosslinkers on the properties of polyimide aerogels, demonstrating that appropriate crosslinking significantly improved the mechanical properties and moisture resistance of aerogels. However, despite the significant progress made with these crosslinkers in relation to improving the performance of polyimide aerogels, their high cost and complex synthesis processes still limit their widespread industrial application [34,35,36].

Herein, we propose a novel synthetic approach by incorporating an Al–O network phase to enhance the thermal and mechanical properties of polyimide aerogels. This addition specifically addresses the limitations of conventional polyimide aerogels, such as poor dimensional stability and insufficient high-temperature mechanical strength. The integration of the alumina network provides a scalable solution, significantly improving the high-temperature performance of the aerogels and enhancing their suitability for aerospace thermal protection applications. The aerogels developed in this study showed remarkable improvements in several key performance areas. After undergoing a 200 °C thermal treatment, the aerogels exhibited only 0.56% linear shrinkage, a significant reduction compared to ~40% shrinkage in conventional polyimide aerogels under similar conditions. This demonstrates their superior dimensional stability at high temperatures. Furthermore, the aerogels maintained a low thermal conductivity of 0.0214 W m^−1^ K^−1^, indicating outstanding thermal insulation properties. The compressive strength of the hybrid polyimide aerogels also showed substantial enhancement. Specifically, the compressive strength increased from 0.265 MPa at room temperature to 0.329 MPa at 200 °C and further to 0.394 MPa at 250 °C, clearly showing improved mechanical strength with rising temperature. To contextualize these advancements, a comparison was made with a pure polyimide aerogel baseline prepared from the same monomer system, as detailed in our prior work [25]. The comparison highlights the clear superiority of the Al–O hybrid aerogels. Most notably, the high-temperature dimensional stability was drastically improved; the thermal shrinkage at 200 °C was reduced from ~5.70% (in the pure polyimide aerogel) to just 0.58% in our PIAs-A0.75 sample, even under a significantly longer thermal exposure (40 min vs. 10 min). Furthermore, significant improvements were also observed in drying shrinkage (reduced from 2.31% to 1.06%) and thermal conductivity (reduced from 0.0238 to 0.0214 W m^−1^ K^−1^). This direct comparison substantiates the efficacy of the Al–O network hybridization strategy in overcoming the key performance limitations of conventional polyimide aerogels. To achieve this, we explored two distinct hybridization strategies: an in situ approach using a sol–gel-derived alumina network (denoted as PIAs-A series) and a direct mixing approach using pre-formed nano-alumina powder (PIAs-a series). A comparative analysis of these two routes was conducted to determine the more effective strategy. Their excellent ability to withstand prolonged thermal exposure confirms the feasibility of introducing Al–O network reinforcement, making these two routes promising candidates for the high-performance thermal protection of the materials used in the aerospace industry.

## 2. Results and Discussion

### 2.1. Preparation Process and Crosslinking Mechanism of the PIAs-A Series

**Figure 1:**
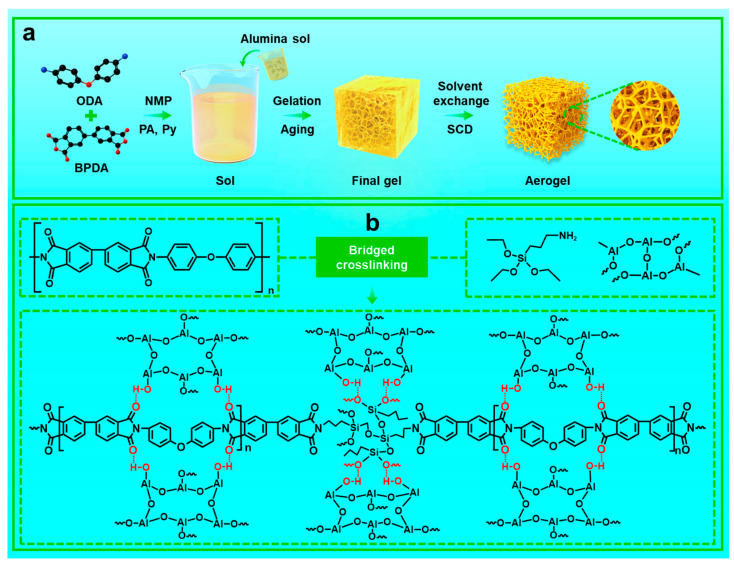
Synthesis and crosslinking mechanisms of PIAs-A aerogels. (**a**) Schematic of the synthesis route for the PIAs-A series. (**b**) Chemical structure and bridged crosslinking mechanism of the PIAs-A series.

The synthetic route to obtain the PIAs-A series is schematically illustrated in Figure 1a. In the selection of diamine and dianhydride monomers, 4,4′-oxidianiline (ODA) and 3,3′, 4,4′-biphenyltetracarboxylic dianhydride (BPDA) were chosen, and a strong polar solvent, N-methylpyrrolidone (NMP), was used to synthesize a polyamide acid (PAA) solution. As a crosslinking agent, 3-aminopropyltriethoxysilane (APTES) was introduced into the acidic PAA solution. Thereafter, alumina sol was added and PAA solution was chemically imidized to form polyimide by adding propionic anhydride as a dehydrating agent and pyridine as a catalyst. During the reaction, the introduced alumina sol strongly interacts with the carbonyl and hydroxyl groups contained in the polyimide chain (Figure 1a) through hydrogen bonding. After forming the initial gel, ethanol was used to replace the residual NMP, propionic anhydride, and pyridine in the initial gel, and finally obtain the polyimide aerogel by supercritical CO_2_-fluid drying (Figure 1a).

It is worth noting that the addition of excessive alumina sol leads to the deterioration of the macroscopic morphology of the polyimide aerogel, which is reflected in an increase in thermal conductivity and bulk density and a decrease in temperature resistance. We believe that the introduction of excess alumina sol will introduce excess water into the PAA solution at the same time, which may lead to the hydrolysis of a dianhydride monomer and the formation of a carboxylic anhydride group, thus affecting the reaction and damaging the stability of the aerogel skeleton structure. As a silane coupling agent, the –NH_2_ group of APTES reacts with anhydride end-caps present in the PAA solution. The subsequent addition of the base catalyst (pyridine) and dehydrating agent then promotes not only the imidization of the polyamic acid, but also the hydrolysis and condensation of the ethoxysilane groups on APTES, ultimately forming a 3D covalently crosslinked network. This process involves two concurrent steps: (1) condensation to establish a covalent backbone, and (2) hydrolysis-polycondensation, ultimately yielding a bridged crosslinked structure (Figure 1b). The choice of aluminum sol is attributed to the high density of surface hydroxyl groups on the in situ-formed alumina network, which, integrated into an internal network of Al–O bonds, coordinate with multiple hydroxyl groups and engage in hydrogen bonding with polyimide chains. This interaction significantly enhances the structural integrity of the polyimide aerogel.

**Figure 2:**
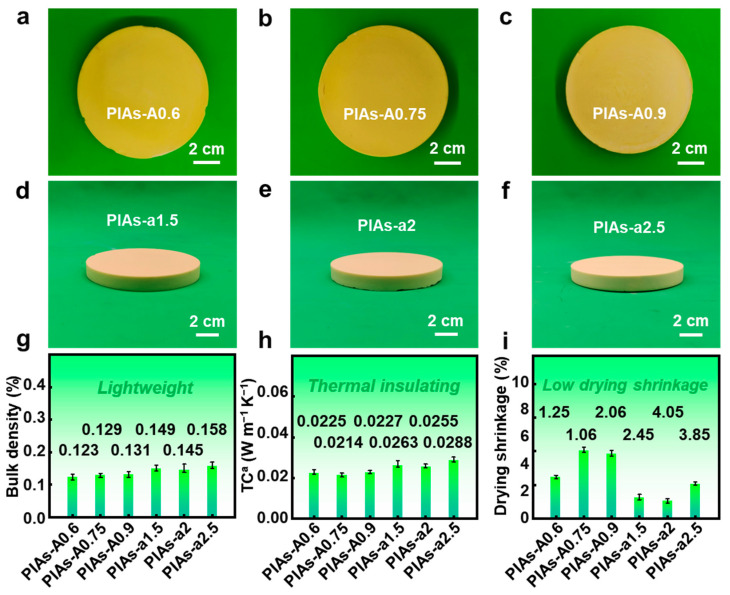
Photographs and physical properties of PIAs-A/a series. Photographs showing formability of (**a**) PIAs-A0.6, (**b**) PIAs-A0.75, (**c**) PIAs-A0.9, (**d**) PIAs-a1.5, (**e**) PIAs-a2, and (**f**) PIAs-a2.5. (**g**) Bulk densities of the PIAs-A/a series. (**h**) Thermal conductivities of the PIAs-A/a series. (**i**) Drying shrinkage of the PIAs-A/a series. Notes: TC^a^ refers to thermal conductivity.

Figure 2a–c displays physical images of the PIAs-A series, with all samples showing excellent formability. Shrinkage during the polyimide aerogel preparation process primarily occurs during the supercritical drying stage. As seen in Figure 2i, the PIAs-A series exhibits relatively minimal linear drying shrinkage, with PIAs-A0.75 showing only 1.06% drying shrinkage, thus enabling the controlled fabrication of these polyimide aerogels. Figure 2g shows that, as the aluminum oxide sol content increases, the bulk density of the polyimide aerogel increases slightly. Room temperature thermal conductivity tests indicate that the aerogels generally have low thermal conductivity, as shown in Figure 2h, with PIAs-A0.75 showing the lowest thermal conductivity (0.0214 W m^−1^ K^−1^), which contributes to its excellent thermal insulation properties. Taking bulk density, thermal conductivity, and drying shrinkage into account, PIAs-A0.75 is the highest-performing material. A key trend observed in Figure 2g is the systematic increase in bulk density with increasing alumina sol content, rising from 0.123 g cm^−3^ for PIAs-A0.6 to 0.149 g cm^−3^ for PIAs-A0.9. This suggests that a higher concentration of the in situ-generated Al–O network leads to a more compact packing of the solid skeleton and a corresponding compaction of the overall nanoporosity.

To compare the effects of different types of aluminum oxide on thermal performance, polyimide aerogels containing nano-aluminum oxide are shown in Figure 2d–f. The experimental results show that the PIAs-a series also exhibits good formability. Figure 2i shows the drying shrinkage rates, and, compared to PIAs-A series, the PIAs-a series experienced greater drying shrinkage during the preparation process, leading to a higher bulk density. As depicted in Figure 2h, the PIAs-a series exhibits a relatively low thermal conductivity at room temperature, which can be attributed to the nanoporosity from the nano-aluminum oxide, which helps to reduce heat transfer through the polyimide aerogels. Increasing the nano-aluminum oxide content leads to minor fluctuations in both thermal conductivity and bulk density.

**Figure 3:**
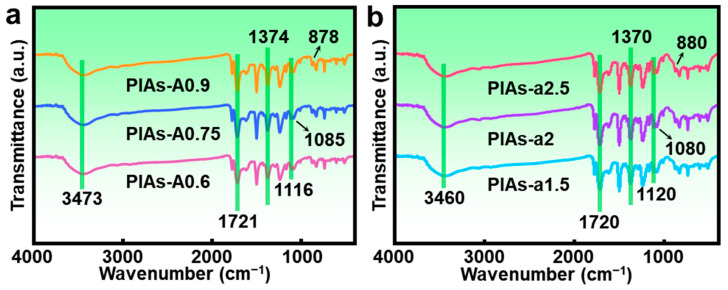
FTIR spectra of the PIAs-A/a series. FTIR spectra of (**a**) the PIAs-A series and (**b**) the PIAs-a series.

The successful synthesis of the polyimide structure was subsequently verified using Fourier transform infrared (FTIR) analysis for both series. The spectra in Figure 3 confirm the formation of the imide ring, showing characteristic peaks near 3470, 1720, and 1370 cm^−1^. A detailed analysis of PIAs-A series identified N–H bond stretching at 3473 cm^−1^, symmetric C=O stretching at 1721 cm^−1^, and C–N stretching of the imide ring at 1374 cm^−1^. The successful introduction of APTES into the polymer backbone was also confirmed by the observation of Si–O bond vibration at 1116 cm^−1^. Both series of polyimide aerogels did not show significant absorption peaks at 1625 cm^−1^, indicating that most of the polyamic acid was imidized, forming long chains composed of phenyl ring structures. The characteristic peaks at 1085 and 878 cm^−1^ in the PIAs-A series correspond to Al–O vibrations, indicating the presence of Al^3+^ and O^2−^ ions on the surface. These results, along with the elemental confirmation by EDS, further confirm the successful preparation of the hybrid polyimide aerogels.

### 2.2. Pore Structure of the PIAs-A Series

A comparison of the physical properties between the PIAs-A and PIAs-a series reveals that the PIAs-A series exhibits a smaller bulk density and drying shrinkage. To further investigate the impact of alumina incorporation on the structure of polyimide aerogels, the analysis below will focus on the performance of PIAs-A0.6, PIAs-A0.75, and PIAs-A0.9.

**Figure 4:**
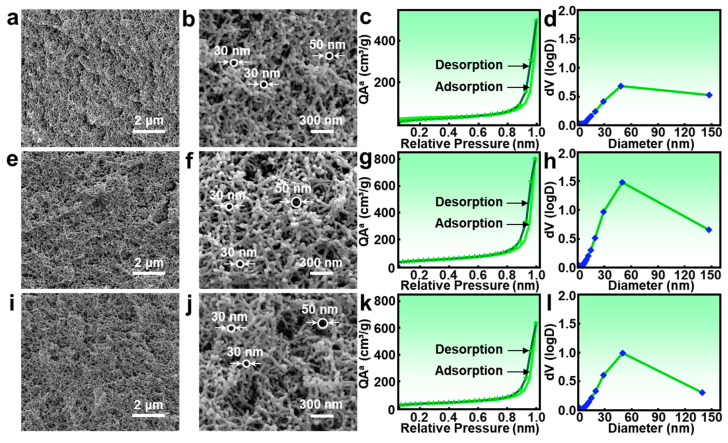
Morphology and nitrogen adsorption–desorption curves of PIAs-A series. FESEM images of (**a**,**b**) PIAs-A0.6, (**e**,**f**) PIAs-A0.75, and (**i**,**j**) PIAs-A0.9. Nitrogen adsorption–desorption isotherms of (**c**) PIAs-A0.6, (**g**) PIAs-A0.75, and (**k**) PIAs-A0.9. Pore size distributions of (**d**) PIAs-A0.6, (**h**) PIAs-A0.75, and (**l**) PIAs-A0.9. Note: QA^a^ refers to quantity adsorbed.

Pore structure was systematically investigated using nitrogen adsorption–desorption analysis and field emission scanning electron microscope (FESEM), as shown in Figure 4. The nitrogen adsorption–desorption isotherms for all samples (Figure 4c,g,k) exhibited a distinct Type IV profile with an H1-type hysteresis loop at high relative pressures. This is a characteristic feature of mesoporous materials with uniform, interconnected pores, which is consistent with the network structure of the aerogel. The sharp uptake in the high-pressure region is due to capillary condensation within the mesopores, further confirming this morphology.

FESEM images provide direct, visual evidence of this structure. High-magnification images (Figure 4a,b,e,f,i,j) reveal that the network is composed of randomly entangled, pearl-chain-like nanoparticles. These images allow for a qualitative visual estimation of the pore sizes; for example, in PIAs-A0.75 (Figure 4f), many pores appear to be in the range of 30–50 nm.

To quantitatively verify these visual observations, the pore size distribution was determined using the Barrett–Joyner–Halenda (BJH) method. The results (Figure 4d,h,l) show that the majority of the pores are concentrated between 10 and 60 nm, with a peak distribution around 30–50 nm. This quantitative data strongly corroborates the visual information from FESEM images, providing a comprehensive and consistent picture of the porous architecture. The data reveal that PIAs-A0.75 exhibits a more uniform pore size distribution compared to PIAs-A0.6 and PIAs-A0.9. This superior pore structure, confirmed by both FESEM and BJH analysis, accounts for its lower thermal conductivity and smaller drying shrinkage. Quantitative analysis of the pore structure confirms that all samples in the PIAs-A series are highly porous. The specific surface areas, as determined using the Brunauer–Emmett–Teller (BET) method, range from 100.4 to 158.2 m^2^ g^−1^, and the total pore volumes range from 0.77 to 1.24 cm^3^ g^−1^. The calculated porosities for all samples are above 89.40%, confirming their rich nanoporous architecture. Notably, the optimal sample, PIAs-A0.75, exhibits the highest specific surface area and total pore volume, which is consistent with its superior thermal and mechanical performance.

### 2.3. High-Temperature Resistance Properties

To evaluate the high-temperature performance of the PIAs-A series, high-temperature tests were conducted on PIAs-A0.6, PIAs-A0.75, and PIAs-A0.9. In the experiment, polyimide aerogel samples were first processed into relatively uniform cubic blocks, and their dimensions and mass were measured before high-temperature treatment. The oven temperature was then raised to 200 °C and stabilized. The polyimide aerogel samples were then placed in a muffle furnace for 2400 s of high-temperature treatment. After the high-temperature test, the samples were immediately removed. Once the aerogels cooled to room temperature, their dimensions and mass were re-measured.

**Figure 5:**
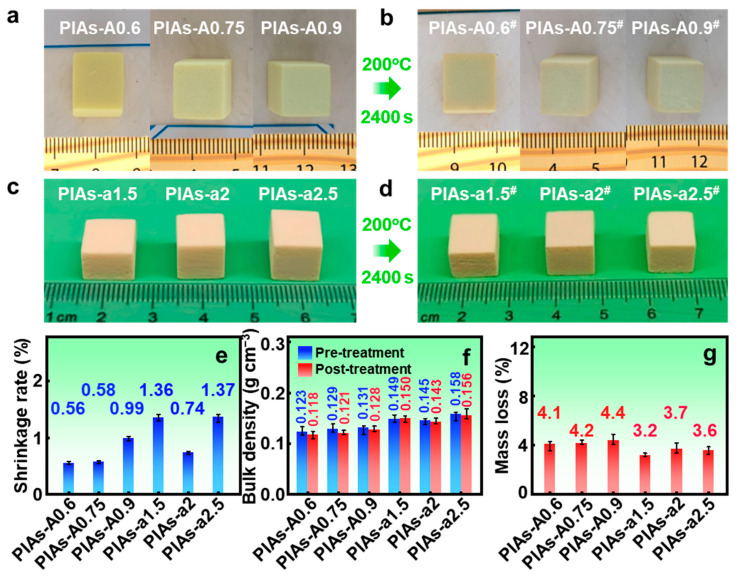
Photographs and physical properties of the PIAs-A/a series before and after the 200 °C thermal treatment. (**a**) Images of the PIAs-A series before the 200 °C treatment. (**b**) Images of the PIAs-A series after the 200 °C treatment. (**c**) Images of the PIAs-a series before the 200 °C treatment. (**d**) Images of the PIAs-a series after the 200 °C treatment. (**e**) Thermal shrinkage for the PIAs-A/a series. (**f**) Bulk densities for the PIAs-A/a series. (**g**) Mass loss for the PIAs-A/a series. Notes: The symbol (#) in (**b**,**d**) stands for samples after 200 °C treatment.

As shown in Figure 5b, after the high-temperature testing, no significant deformation or cracking was observed in the PIAs-A0.6, PIAs-A0.75, or PIAs-A0.9 sample, indicating that the PIAs-A series of aerogels possess excellent thermal stability. To evaluate their high-temperature stability more quantitatively, the thermal shrinkage rate was a key parameter. Figure 5e presents the thermal shrinkage data of the polyimide aerogels after 200 °C treatment. The results show that the thermal shrinkage rates of the PIAs-A series are all below 1.00%, with PIAs-A0.6 and PIAs-A0.75 exhibiting particularly low shrinkage rates. This phenomenon can be attributed to the robust nanonetwork structures in PIAs-A0.6 and PIAs-A0.75, which effectively suppress the free movement of polyimide molecular chains, thereby reducing thermal shrinkage. Moreover, the Al–O network structure present in PIAs-A0.6 and PIAs-A0.75 helps to dissipate thermal stress during thermal treatment, further reducing the thermal shrinkage rate. Therefore, the PIAs-A0.6 and PIAs-A0.75 samples exhibit superior stability and resistance to thermal shrinkage under high-temperature conditions.

To compare the high-temperature performance of the PIAs-A and PIAs-a series, high-temperature tests were also performed on PIAs-a1.5, PIAs-a2, and PIAs-a2.5, with thermal treatment at 200 °C for 2400 s. As shown in Figure 5d, after high-temperature treatment, no noticeable shrinkage or cracking was observed in the PIAs-a series, indicating their excellent thermal stability. As shown in Figure 5c–e, the thermal shrinkage rate of the PIAs-a series was limited to a maximum of only 1.37%, further illustrating the reinforcement of their nanonetwork structure. Furthermore, the introduction of nano-alumina creates structural reinforcement points within nanoporous network, which effectively restricts molecular chain movement and enhances thermal stability through interfacial interactions.

After the 200 °C thermal treatment, a slight but consistent decrease in bulk density was observed for all samples (Figure 5f). This phenomenon is the net result of two competing effects. The dominant effect at this temperature is a mass loss of approximately 4% (Figure 5g), which is attributed to the evaporation of physically adsorbed water from the high-surface-area network of the aerogel. Concurrently, the robust hybrid network undergoes only a very small volume shrinkage (corresponding to a linear shrinkage of ~1%, Figure 5e). Because the percentage of mass loss is greater than the percentage of volume shrinkage, the overall bulk density slightly decreases after the 200 °C treatment. The results of the high-temperature tests on both the PIAs-A and PIAs-a series indicate that the incorporation of aluminum oxide enhanced the thermal resistance of the polyimide aerogels. The Al–O network structure imparts thermal stability to the polyimide molecular chains and suppresses high-temperature shrinking of the aerogel network. Notably, the thermal shrinkage rate of the PIAs-A series is lower than that of the PIAs-a series, providing a foundation for further investigation to improve the high-temperature performance of polyimide aerogels.

**Figure 6:**
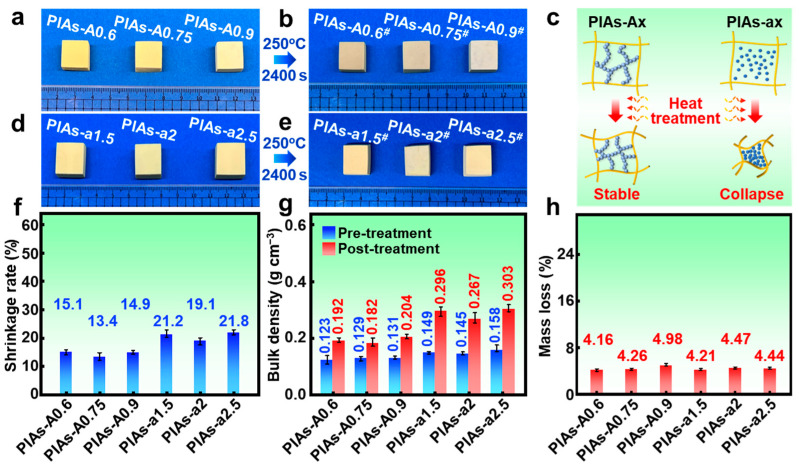
Photographs and physical properties of the PIAs-A/a series before and after the 250 °C thermal treatment. (**a**) Images of the PIAs-A series before the 250 °C treatment. (**b**) Images of the PIAs-A series after the 250 °C treatment. (**c**) Schematic showing the differences in the thermal shrinkage rates between the PIAs-A series and the PIAs-a series. (**d**) Images of the PIAs-a series before the 250 °C thermal treatment. (**e**) Images of the PIAs-a series after the 250 °C thermal treatment. (**f**) Comparison of thermal shrinkage rate for the PIAs-A/a series. (**g**) Comparison of bulk densities for the PIAs-A/a series. (**h**) Comparison of mass loss for the PIAs-A/a series. Notes: The symbol (#) in (**b**,**e**) stands for samples after 250 °C treatment.

High-temperature tests were then performed at 250 °C to further investigate the thermal performance of the polyimide aerogels for their application as thermal protection materials in aerospace. As shown in Figure 6a,b, after 2400 s of thermal treatment at 250 °C, no macroscopic cracking or sintering was observed in the PIAs-A series, further confirming their excellent high-temperature resistance. Following the 250 °C thermal treatment, the PIAs-A series exhibited a low mass loss rate (at most 4.98%), showing only a slight decrease compared to the aerogels treated at 200 °C. This result demonstrates the outstanding thermal stability of the polyimide aerogels. As shown in Figure 6g, the bulk densities of the PIAs-A series increase after 250 °C treatment, mainly due to insufficient dimensional stability at higher temperatures, leading to more significant volume shrinkage. Figure 6f reveals that the thermal shrinkage rates of PIAs-A0.6, PIAs-A0.75, and PIAs-A0.9 were 15.10%, 13.40%, and 14.90%, respectively. Compared to the thermal shrinkage rates observed after the 200 °C high-temperature exposure, these values show a notable increase. The Si–O bonds in APTES form a network with the polyimide main chain, and the introduction of the Al–O network structure further strengthens this nanonetwork.

However, the connections between the polyimide molecular chains are not sufficiently strong, and, at this high temperature, they are unable to effectively restrain the movement of the molecular chains. Nevertheless, compared to pure polyimide aerogels, which exhibit a thermal shrinkage rate of ~40% at 250 °C, the PIAs-A series demonstrates significantly improved high-temperature performance. Similarly, the PIAs-a series underwent high-temperature tests at 250 °C under the same thermal treatment conditions and duration as the PIAs-A series. As shown in Figure 6d,e, the PIAs-a series exhibited significant thermal shrinkage and slight deformation in appearance after the 250 °C high-temperature test. This result suggests that the network structure of the PIAs-a series is less thermally stable compared to that of the PIAs-A series. This difference may be attributed to the fact that, while nano-alumina provides porosity, reducing the thermal conductivity of the polyimide aerogel, it does not form a self-supporting and integrated network structure within the polyimide skeleton. As schematically illustrated in Figure 6c, unlike the in situ-formed alumina sol network, nano-alumina particles do not offer the same structural support to the polyimide skeleton at high temperatures. The loose distribution of nano-alumina in the aerogel network leads to the significant collapse of the pores during shrinkage, resulting in noticeable volume reduction at the macroscopic level.

In contrast, PIAs-A series, which incorporates alumina sol, benefits from a secondary network structure strengthened by the in situ-formed alumina sol network, greatly enhancing its high-temperature performance. Figure 6f shows the thermal shrinkage rates of the PIAs-a series after the 250 °C high-temperature test, with an average value of approximately 20%. However, compared to pure polyimide aerogels, the temperature resistance of the PIAs-a series has also improved to some extent. As shown in Figure 6h, the PIAs-a series also exhibits a relatively low mass loss, primarily due to the intrinsic excellent thermal stability of polyimide. The bulk density increased by nearly 50%, which is a result of the significant thermal shrinkage observed in the PIAs-a series at high temperatures. With the high-temperature tests on both the PIAs-A and PIAs-a series, it is evident that the introduction of Al–O network structure improves the thermal resistance of polyimide aerogels. Alumina helps to alleviate thermal stress within the aerogel network, enhancing its high-temperature dimensional stability. Among the two series, the PIAs-A series exhibited superior high-temperature performance.

To further confirm and quantify the elemental composition, particularly the aluminum content, energy dispersive X-ray spectroscopy (EDS) analysis was performed. The results are summarized in Table 1. The data clearly confirms that the aluminum (Al) element was successfully incorporated into all hybrid aerogel skeletons. For the PIAs-A series, the measured Al content (wt.%) systematically increased with the sol addition amount (PIAs-A0.6: 0.45% < PIAs-A0.75: 0.57% < PIAs-A0.9: 0.80%), which validates the controllability of our experimental design. Furthermore, as expected, the PIAs-a series, which used 100% solid powder, shows a significantly higher “real aluminum content” (e.g., PIAs-a2: 6.61 wt.%). Most importantly, by comparing the samples before (e.g., PIAs-a2.5: 2.67 wt.%) and after heat treatment (e.g., PIAs-a2.5*−200 °C: 2.60 wt.%), the EDS data indicates that the Al element content showed almost no significant change. This provides critical chemical evidence for the thermal stability of the Al-O network, supporting the macroscopic dimensional stability previously observed.

### 2.4. Thermal Insulation Performance of the PIAs-A Series

**Figure 7:**
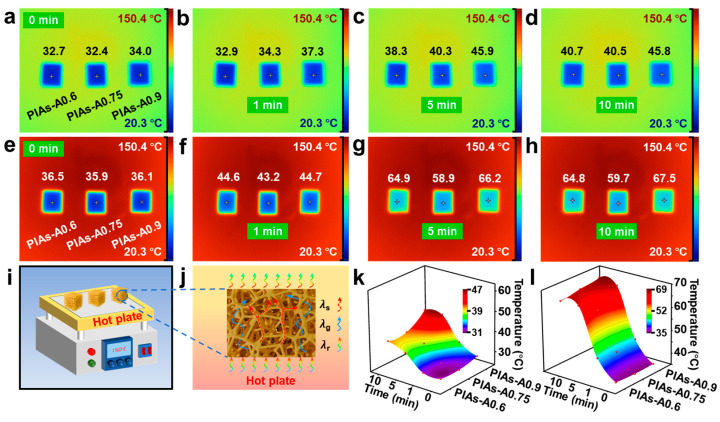
Thermal insulation performance of the PIAs-A series. Thermographic images of the PIAs-A series on (**a**–**d**) the 100 °C hot-plate and (**e**–**h**) the 150 °C hot-plate. (**i**) Schematic of the thermal insulation test. (**j**) Schematic of the heat transfer mechanism. (**k**) Cold-side temperature diagram of the 100 °C hot-plate. (**l**) Cold-side temperature diagram of the 150 °C hot-plate.

To quantitatively evaluate the thermal insulation performance of the polyimide aerogels, tests were performed on the PIAs-A series, as their thermal resistance significantly surpassed that of the PIAs-a series. Samples of PIAs-A0.6, PIAs-A0.75, and PIAs-A0.9 were placed horizontally on a hot-plate with temperature control. The side in contact with the heat source was termed as the ‘hot side’, while the opposite surface was referred to as the ‘cold side’. A thermal imaging camera was employed to record the temperature changes on the cold side. The platform was first set to 100 °C, reaching an actual temperature of 100.7 °C. When the polyimide aerogel samples were placed on the platform, measurements began, and the cold-side temperature was recorded using an infrared thermal imaging camera. As shown in Figure 7a, the initial cold-side temperatures for PIAs-A0.6, PIAs-A0.75, and PIAs-A0.9 were 32.7, 32.4, and 34.0 °C, respectively. Over time, the cold-side temperature gradually increased. After 5 min, a thermal equilibrium was achieved, and the cold-side temperatures stabilized. The criterion for reaching thermal equilibrium was defined as the point at which the rate of temperature change on the cold side became negligible. As observed in the thermographic images from the 150 °C test (Figure 7h), the cold-side temperature for PIAs-A0.75 was 58.9 °C at the 5 min mark and 59.7 °C at the 10 min mark. The minimal temperature increase of only 0.8 °C over this subsequent 5 min interval confirms that the heat flow had effectively stabilized and that the system had reached a state of thermal equilibrium. Once the thermal equilibrium was reached, the cold-side temperatures were recorded as 40.7, 40.5, and 45.8 °C for PIAs-A0.6, PIAs-A0.75, and PIAs-A0.9, respectively (Figure 7d). These results demonstrate the excellent thermal insulation performance of the PIAs-A series. The thermal insulation performance of PIAs-A0.75 was notably superior to that of PIAs-A0.6 and PIAs-A0.9, primarily due to its more uniform and abundant nanopores, which effectively hindered heat transfer.

Using the same testing procedure, the temperature of the hot-plate was set to 150 °C, with an actual temperature of 148.7 °C. As shown in Figure 7e, when the samples were placed horizontally on the hot-plate, the initial cold-side temperatures of PIAs-A0.6, PIAs-A0.75, and PIAs-A0.9 were 36.5, 35.9, and 36.1 °C, respectively. The cold-side temperature increased over time. After 5 min, the cold-side temperatures of PIAs-A0.6, PIAs-A0.75, and PIAs-A0.9 were 64.9, 58.9, and 66.2 °C, respectively. Using an infrared thermal camera to monitor the temperature changes, it was observed that, after 10 min, the heat flow reached equilibrium, and the cold-side temperature stabilized. Figure 7h shows that, after 10 min, the final steady state cold-side temperatures of PIAs-A0.6, PIAs-A0.75, and PIAs-A0.9 were 64.8, 59.7, and 67.5 °C, respectively.

The temperature difference between the hot and cold sides of the polyimide aerogels reached nearly 90 °C, indicating that the thermal insulation performance of the PIAs-A series became more pronounced at higher temperatures. Figure 7k,l shows temperature-rise diagrams for the 100 and 150 °C hot-plate tests; among the PIAs-A series, PIAs-A0.75 exhibited superior thermal insulation compared to PIAs-A0.6 and PIAs-A0.9 at different heat-source temperatures, which also explains why PIAs-A0.75 has a lower thermal shrinkage rate. The excellent thermal insulation performance is attributed to the polyimide aerogel network structure. The thermal conductivity of aerogel is mainly composed of solid thermal conductivity (λ_s_), gas thermal conductivity (λ_g_), and radiation thermal conductivity (λ_r_). As illustrated in Figure 7j, its rich pore structure forms an extremely tortuous thermal conduction path, which significantly impedes heat transfer and gives the polyimide aerogel its outstanding thermal insulation properties.

### 2.5. Enhanced Compressive Strength at Elevated Temperatures

**Figure 8:**
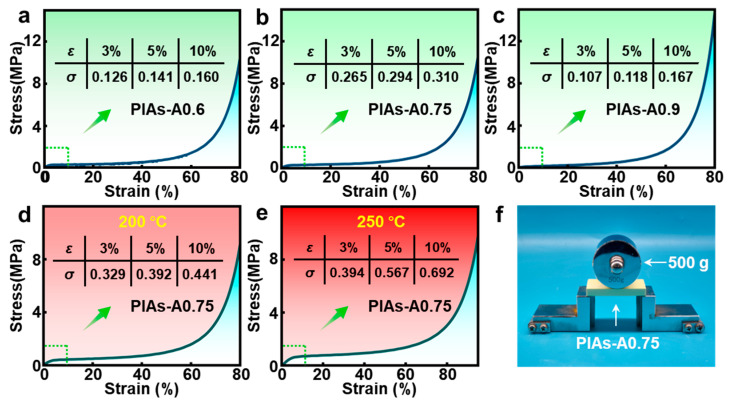
Compressive properties and toughness of the PIAs-A series. (**a**–**c**) Compression stress–strain curves of PIAs-A0.6, PIAs-A0.75, and PIAs-A0.9. (**d**) Compression stress–strain curve of PIAs-A0.75 after thermal treatment at 200 °C. (**e**) Compression stress–strain curve of PIAs-A0.75 after thermal treatment at 250 °C. (**f**) Photograph demonstrating the toughness of PIAs-A0.75.

To investigate the mechanical properties of polyimide aerogels, compression tests were conducted on both the PIAs-A and PIAs-a series. As shown in Figure 8a–c, PIAs-A0.75 in the PIAs-A series exhibited the best compression performance. With increasing alumina sol content, the compression performance of PIAs-A series first improved and then declined. This trend is primarily attributed to excessive alumina sol causing the polyimide aerogel nanonetwork to aggregate into particulate clusters, leading to increased brittleness and reduced compressive strength at the macroscopic level.

To study the mechanical strength of the PIAs-A series aerogels after high-temperature treatment, compression tests were performed on the optimal PIAs-A0.75 samples post-thermal treatment. Figure 8d illustrates the compressive performance of PIAs-A0.75 after thermal treatment at 200 °C for 2400 s. At a compressive strain of 3%, the compressive stress reached 0.329 MPa, representing a significant increase from the original compressive stress of 0.265 MPa for PIAs-A0.75. This pronounced enhancement in mechanical properties is a direct macroscopic manifestation of a more robust and stable underlying network. Crucially, this strengthening is not due to crystallization, as the treatment temperatures (from 200 to 250 °C) are well below the glass transition temperature of the rigid polyimide backbone, meaning that the chains lack the mobility to form an ordered crystalline structure. Instead, the enhancement is attributed to nanoscale phenomena within the amorphous network, primarily the further condensation of the inorganic Al-O network and the relaxation of internal stresses, which collectively create a more effectively crosslinked and stable skeletal structure. Figure 8e shows the compressive performance of PIAs-A0.75 after thermal treatment at 250 °C. Despite the significant thermal shrinkage, the increased bulk density resulted in higher compressive strength. This finding suggests that the network structure of PIAs-A0.75 remained largely intact, even after the 250 °C heat test.

To further evaluate the compressive performance, a test setup was arranged, as shown in Figure 8f. A sample of PIAs-A0.75 was placed horizontally between two L-shaped iron frames with a span of 50 mm. A 500 g weight is placed sideways against the center of the aerogel, such that a concentrated bending force was applied to the center. The aerogel edges are placed on the frame to maximize force arm applied by the weight. The PIAs-A0.75 sample shows excellent toughness, without any signs of fracture or obvious deformation.

### 2.6. Flame Retardancy and Thermal Stability

**Figure 9:**
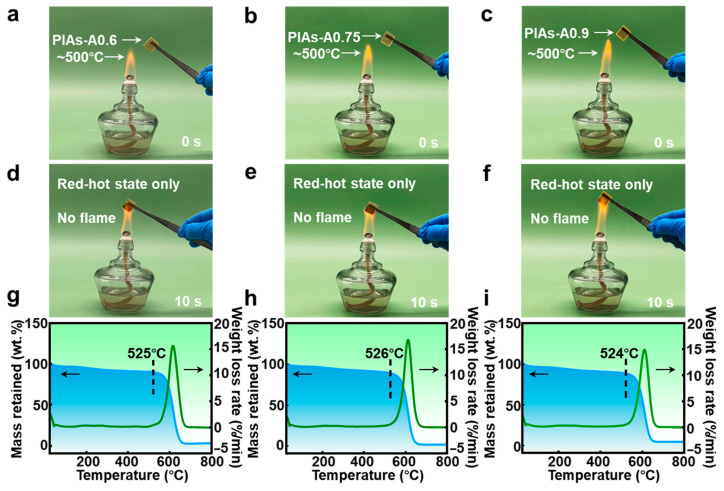
Flame retardancy and thermal stability of the PIAs-A series. Photographs of (**a**,**d**) PIAs-A0.6, (**b**,**e**) PIAs-A0.75, and (**c**,**f**) PIAs-A0.9 burning on the outer flame of an alcohol burner. TG-DTG curves of (**g**) PIAs-A0.6, (**h**) PIAs-A0.75, and (**i**) PIAs-A0.9.

To investigate the flame-retardant and fire-resistant properties of the polyimide aerogels, PIAs-A0.6, PIAs-A0.75, and PIAs-A0.9 were processed into standard small cubic samples. The small polyimide aerogel cubes were then exposed to the outer flame of an alcohol burner (with an outer flame temperature of approximately 500 °C) to test their flame-retardant performance. Figure 9d–f shows the combustion behavior of PIAs-A0.6, PIAs-A0.75, and PIAs-A0.9. Upon contact with the outer flame of the alcohol burner, the polyimide aerogel cubes exhibit only partial volume shrinkage and begin to glow red, with no obvious visible combustion flames. This observation indicates that the PIAs-A series possesses inherent flame-retardant properties.

The flame-retardant performance of polyimide aerogels is mainly attributed to their molecular structure. The PIAs-A series contains numerous aromatic ring structures which that C–C and C–H bonds. These bonds have high dissociation energies, making the polyimide aerogel molecular structure resistant to thermal cleavage and combustion at high temperatures. Additionally, the presence of C=O bonds in the molecular structure of the PIAs-A series contributes to its flame resistance. These bonds can promote the formation of a stable char layer during combustion, which suppresses the combustion reaction. Consequently, the PIAs-A series exhibits excellent flame-retardant and fire-resistant performance. Moreover, the nanoporous network structure of the PIAs-A series also imparts flame-retardant properties. At high temperatures, the nanoporous network structure can inhibit the spread of flames and limit oxygen diffusion, further enhancing the combustion resistance of the aerogels. This combination of molecular and structural characteristics makes the PIAs-A series highly effective as a flame-retardant material.

Excellent thermal stability is a crucial property for thermal protection materials used in aerospace applications. To investigate the thermal stability of the PIAs-A series, thermogravimetric analysis (TG) and derivative thermogravimetry (DTG) were conducted under an air atmosphere, measuring the mass evolution of polyimide aerogels from room temperature to 800 °C. Figure 9g–i displays the TG-DTG curves of PIAs-A0.6, PIAs-A0.75, and PIAs-A0.9. In the first stage, from room temperature to approximately 300 °C, the TG curves of all three samples showed a slight decrease in mass. This initial weight loss was likely due to the evaporation of absorbed water and the decomposition of residual solvents within the porous structure. As the temperature increased beyond 500 °C, the TG curves of the PIAs-A series began to exhibit a sharp decline. This rapid weight loss can be attributed to the decomposition of the polymer molecular chains and the breaking of weak bonds in PIAs-A0.6, PIAs-A0.75, and PIAs-A0.9. The DTG curves reveal distinct endothermic peaks around 600 °C for all three polyimide aerogels, corresponding to the thermal decomposition process. As shown in Figure 9g–i, the maximum thermal decomposition rates of PIAs-A0.6, PIAs-A0.75, and PIAs-A0.9 occurred at 525, 526, and 524 °C, respectively. This quantitatively determined decomposition temperature provides a clear scientific explanation for the phenomenon observed in the preceding flame test. The test, conducted in an alcohol burner flame of approximately 500 °C, operates just below this critical decomposition threshold. This explains why the material remains structurally stable enough to exhibit a “red-hot state only” rather than active combustion, demonstrating a strong consistency between the two characterization methods. Among these samples, PIAs-A0.75 exhibited the highest thermal decomposition temperature. This can be credited to its stable molecular structure and uniform nanoporous architecture, which renders the overall network structure more robust. Moreover, the polyimide molecular chains of PIAs-A0.75 are connected to abundant benzene rings, enhancing its intermolecular crosslinking interactions. The molecular structure of the PIAs-A series also contains a high density of aromatic rings, whose π-π stacking interactions further reinforce intermolecular forces. This reinforcement contributes to the improved thermal stability of the polyimide aerogels in the PIAs-A series.

## 3. Conclusions

Polyimide aerogels were successfully reinforced using the integration of an Al–O network formed via an in situ alumina sol, significantly enhancing their dimensional stability and thermal insulation under elevated temperatures. The optimized aerogel (PIAs-A0.75) exhibited a uniform nanoporous architecture (pore sizes predominantly in the range of 10–60 nm), ultralow density (0.129 g cm^−3^), and minimal thermal conductivity (0.0214 W m^−1^ K^−1^). After heating at 200 °C for 2400 s, the resulting PIAs-A series exhibited exceptional dimensional stability with linear shrinkage as low as 0.56%, markedly outperforming pure polyimide aerogels. Even at a more challenging temperature of 250 °C, shrinkage remained low (13.40%), confirming the efficacy of the Al–O network reinforcement. Mechanical evaluations indicated improved compressive strength (0.329 MPa at 3% strain after 200 °C treatment), while thermal insulation tests demonstrated superior performance, maintaining a cold-side temperature of 59.7 °C under a 150 °C heat source. Additionally, the PIAs-A series exhibited inherent flame retardancy, resisting ignition upon direct flame exposure. Overall, the incorporation of the Al–O phase substantially advanced the thermal stability, mechanical robustness, and fire retardancy of polyimide aerogels, highlighting their promising utility as thermal protection materials in aerospace and related extreme environments. Future work will focus on evaluating their long-term performance under cyclic thermal loading and expanding the mechanical characterization to further validate their suitability for demanding aerospace applications.

## 4. Materials and Methods

### 4.1. Materials

All reagents were used as received without requiring further purification. The monomers for the polyimide synthesis, namely 4,4′-Oxidianiline (ODA, 98%) and 3,3′,4,4′-biphenyltetracarboxylic dianhydride (BPDA, 97%), were purchased from Shanghai Aladdin Biochemical Technology Co., Ltd., China. The same company also supplied the solvent N-methylpyrrolidone (NMP), the catalysts propionic anhydride (PA) and pyridine (Py), and acetic acid. Ethyl alcohol (EtOH, analytical reagent) was a product of Shanghai Titan Scientific Co., Ltd., China. Finally, aluminum isopropoxide (AIP) and 3-aminopropyltriethoxysilane (APTES) were purchased from Macklin Co., Ltd., Shanghai, China. The nano-alumina powder used for the PIAs-a series was also procured from Shanghai Aladdin Biochemical Technology Co., Ltd., China. According to the supplier, the powder is α-phase Al_2_O_3_ with a purity of 99.99%, an average particle size of 20–30 nm, and a specific surface area of 50 m^2^ g^−1^.

### 4.2. Fabrication Process of High-Temperature-Resistant Polyimide Aerogels

For a typical synthesis of the optimal sample, PIAs-A0.75, 3.94 g of ODA was first dissolved in 98.00 mL of NMP in a flask under magnetic stirring. After the ODA was completely dissolved, 5.88 g of BPDA was added to the solution, corresponding to an ODA:BPDA molar ratio of approximately 60:61 and resulting in a polyamic acid (PAA) solution with a final total solid content of approximately 10 wt%. Subsequently, 10.33 mL of propionic anhydride (PA) and 6.58 mL of pyridine (Py) were added as the imidization agent and catalyst, respectively. After stirring for 30 min, 1.30 g of APTES was introduced. Finally, 0.75 g of the prepared alumina sol was added to the mixture, and the resulting solution was stirred for another 20 min before being poured into molds. The aluminum oxide sol was synthesized by combining water, acetic acid, and AIP in a molar ratio of 1:0.16:0.03. After stirring for 20 min, the polyimide solution was poured into molds and held in an oven at 25 °C. After gelation, a polyimide initial gel was formed. To prevent cracking, anhydrous ethanol was used for liquid sealing of the initial gel. The initial gel underwent aging and solvent exchange. In this work, aging was conducted in a 75 °C water bath, while solvent exchange was carried out using anhydrous ethanol, with a replacement frequency of 3–4 h per exchange and a total replacement period of 2–3 d. After the aging and solvent exchange, the final gel was obtained. The final gel was then dried using supercritical CO_2_-fluid drying at 60 °C and 12.5 MPa for 8 h to yield the polyimide aerogel.

Two experimental groups were prepared based on the type of alumina introduced. The samples are named based on the type and absolute mass of the alumina additive used in the synthesis. The series with alumina sol is denoted as PIAs-Ax, where x represents the mass in grams (g) of the alumina sol solution added. The sol has a general chemical formula of a (AlO·nH_2_O)·bHAc and a solid content of approximately 10%. For example, PIAs-A0.75 was prepared using 0.75 g of this sol. The series with nano-alumina powder is denoted as PIAs-ax, where x represents the mass in grams (g) of the added nano-alumina powder. For example, PIAs-a1.5 was prepared using 1.5 g of nano-alumina. A direct comparison of equal masses would be scientifically misleading, given that the sol only has a 10% solid content while the nanopowder is a concentrated solid. Therefore, the more rigorous approach was to treat the “in situ sol–gel method” (A-series) and the “direct powder addition method” (a-series) as two distinct hybridization strategies. The different mass ranges for the two series were chosen to independently explore the optimal formulation for each strategy. Consequently, the comparisons between the A- and a-series in this study are intended to evaluate the optimal performance achievable by each hybridization route.

### 4.3. Characterizations

The morphology and microstructure of the PIAs-A series were examined using a field emission scanning electron microscope (FESEM, FEI Inspect F50, Eindhoven, The Netherlands). The elemental composition of the aerogels was analyzed using energy dispersive X-ray spectroscopy (EDS), which was performed using the same instrument as the FESEM. The pore size distribution and the specific surface area were determined by the Barrett–Joyner–Halenda (BJH) method and Brunauer–Emmett–Teller (BET) method using a Quadra Sorb SI analyzer (Quantachrome Instruments, Boynton Beach, FL, USA). To confirm the chemical structures, Fourier transform infrared (FTIR) spectra of both PIAs-A and PIAs-a series were recorded on a Thermo Scientific Nicolet iS5 spectrophotometer (Waltham, MA, USA); the samples were prepared as KBr pellets and scanned over a range of 400–4000 cm^−1^. Thermal conductivity was measured using cylindrical samples (Φ 39 mm × 15 mm) using a TC3000E apparatus (Xi’an Xiatech Electronics Co., Ltd., Xi’an, China). The thermal stability of PIAs-A was investigated by thermogravimetric analysis (TG) and derivative thermogravimetry (DTG) using a HCT-3 analyzer (Beijing Henven Scientific Instrument Co., Ltd., Beijing, China), heating the samples from 35 °C to 800 °C at a rate of 10 °C min^−1^ in an air atmosphere. A custom-built setup was assembled to evaluate the thermal insulation performance, which consisted of a thermostat water bath (DF101S, Gongyi Yuhua Instrument Co., Ltd., Gongyi, China), an infrared thermal imager (Fluke Tis 60+, Fluke, Everett, WA, USA), and a Sony camera (α7 II, Sony, Tokyo, Japan). Additionally, the self-extinguishing behavior of PIAs-A series was tested using an alcohol burner. Mechanical performance was assessed using a microcomputer-controlled electronic universal testing machine (XBD4204, Jinan Kexin Testing Machine Co., Ltd., Jinan, China), during which the bottom platen remained stationary while the top platen descended at a constant speed of 0.5 mm min^−1^. The bulk density (ρ) of the aerogel samples was determined by the mass/volume method. The mass (m) of each sample was measured using a high-precision analytical balance (0.1 mg precision). The dimensions were measured at five different positions using a digital caliper, and the average values were used to calculate the volume (V). The bulk density was then calculated using Equation (1):

(1) $$\rho =\;\;\frac{m}{V}$$

The porosity of aerogels was calculated using Equation (2):

(2) $$Porosity=\left(1-\frac{\rho }{\rho_{s}}\right)\times 100\%$$

where ρ and $\rho_{s}$ are the bulk density and skeletal density of the aerogels, respectively.

## Figures and Tables

**Table 1:** EDS analysis of PIAs-A/a series.

Samples	C (wt.%)	N (wt.%)	O (wt.%)	Al (wt.%)	Si (wt.%)	C (at.%)	N (at.%)	O (at.%)	Al (at.%)	Si (at.%)
PIAs-A0.6	74.04	5.46	19.83	0.45	0.23	78.84	4.99	15.85	0.21	0.10
PIAs-A0.75	72.86	6.52	19.84	0.57	0.21	77.77	5.97	15.9	0.27	0.10
PIAs-A0.9	74.83	5.87	18.14	0.80	0.36	79.62	5.35	14.49	0.38	0.16
PIAs-A0.75 *−200 °C	73.36	6.27	19.71	0.48	0.19	78.19	5.73	15.77	0.23	0.09
PIAs-a1.5	64.31	4.31	24.76	6.21	0.42	71.83	4.12	20.77	3.09	0.20
PIAs-a2	63.77	4.23	25.07	6.61	0.33	71.41	4.06	21.08	3.29	0.16
PIAs-a2.5	72.28	5.51	19.16	2.67	0.38	77.94	5.10	15.51	1.28	0.17
PIAs-a2.5 *−200 °C	74.56	4.35	18.07	2.60	0.43	80.01	4.00	14.56	1.24	0.20

**Notes:** The asterisk (*) in Table 1 stands for samples after 200 °C treatment.

## Data Availability

The data that support the findings of this study are available from the corresponding author upon reasonable request.

## References

[1] Lee C., Chen S. (2019). Recent progress in the study of transition in the hypersonic boundary layer. Natl. Sci. Rev..

[2] Sun B., Oran E. (2018). New principle for aerodynamic heating. Natl. Sci. Rev..

[3] Zhao S., Siqueira G., Drdova S., Norris D., Ubert C., Bonnin A., Galmarini S., Ganobjak M., Pan Z., Brunner S. (2020). Three-dimensional printing of silica aerogels. Nature.

[4] Wang X., Chen X., He Q., Hui Y., Xu C., Wang B., Shan F., Zhang J., Shao J. (2024). Bidirectional, multilayer MXene/Polyimide aerogels for ultra-broadband microwave absorption. Adv. Mater..

[5] Liu C., Wang M., Wang J., Xu G., Zhang S., Ding F. (2024). Double-phase-networking polyimide hybrid aerogel with exceptional dimensional stability for superior thermal protection system. Small.

[6] Pierre A.C., Pajonk G.M. (2002). Chemistry of aerogels and their applications. Chem. Rev..

[7] Cheng Y., Zhang X., Qin Y., Dong P., Yao W., Matz J., Ajayan P.M., Shen J., Ye M. (2021). Super-elasticity at 4 K of covalently crosslinked polyimide aerogels with negative Poisson’s ratio. Nat. Commun..

[8] Yang Y., Yang Y., Ye L., Zhao T., Li H. (2024). Microphase separation regulation of polyurethane modified phenolic resin aerogel to enhance mechanical properties and thermal insulation. Chem. Eng. J..

[9] Chidambareswarapattar C., McCarver P.M., Luo H., Lu H., Sotiriou-Leventis C., Leventis N. (2013). Fractal Multiscale Nanoporous Polyurethanes: Flexible to Extremely Rigid Aerogels from Multifunctional Small Molecules. Chem. Mater..

[10] Cai J., Kimura S., Wada M., Kuga S., Zhang L. (2008). Cellulose aerogels from aqueous alkali hydroxide–urea solution. ChemSusChem.

[11] Chidambareswarapattar C., Xu L., Sotiriou-Leventis C., Leventis N. (2013). Robust Monolithic Multiscale Nanoporous Polyimides and Conversion to Isomorphic Carbons. RSC Adv..

[12] Liu C., Wang M., Zhao X., Yang C., Wei R., Zeng W., Ding F., Zhang S., Lei Y. (2025). High-Temperature Resistant Polyimide Aerogels with Extreme Condition Tolerance Constructed by In Situ Skeleton Encapsulation Growth. Adv. Funct. Mater..

[13] Zhao X., Ruan K., Qiu H., Zhong X., Gu J. (2023). Fatigue-resistant polyimide aerogels with hierarchical cellular structure for broadband frequency sound absorption and thermal insulation. Adv. Compos. Hybrid Mater..

[14] Ghaffari-Mosanenzadeh S., Tafreshi O.A., Karamikamkar S., Saadatnia Z., Rad E., Meysami M., Naguib H.E. (2022). Recent advances in tailoring and improving the properties of polyimide aerogels and their application. Adv. Colloid Interface Sci..

[15] Wang S., Ding R., Liang G., Zhang W., Yang F., Tian Y., Yu J., Zhang S., Ding B. (2024). Direct Synthesis of Polyimide Curly Nanofibrous Aerogels for High-Performance Thermal Insulation Under Extreme Temperature. Adv. Mater..

[16] Qiao S., Kang S., Zhu J., Wang Y., Yu J., Hu Z. (2021). Facile strategy to prepare polyimide nanofiber assembled aerogel for effective airborne particles filtration. J. Hazard. Mater..

[17] Meador M.A.B., Malow E.J., Silva R., Wright S., Quade D., Vivod S.L., Guo H., Guo J., Cakmak M. (2012). Mechanically strong, flexible polyimide aerogels cross-linked with aromatic triamine. ACS Appl. Mater. Interfaces.

[18] Wordsworth R., Kerber L., Cockell C. (2019). Enabling Martian habitability with silica aerogel via the solid-state greenhouse effect. Nat. Astron..

[19] Liu H., Chen X., Zheng Y., Zhang D., Zhao Y., Wang C., Pan C., Liu C., Shen C. (2021). Lightweight, superelastic, and hydrophobic polyimide nanofiber/MXene composite aerogel for wearable piezoresistive sensor and oil/water separation applications. Adv. Funct. Mater..

[20] Yao K., Song C., Fang H., Wang F., Chen L., Jiang S., Zhang G., Hou H. (2023). Freezing-extraction/vacuum-drying method for robust and fatigue-resistant polyimide fibrous aerogels and their composites with enhanced fire retardancy. Engineering.

[21] Tian J., Yang Y., Xue T., Chao G., Fan W., Liu T. (2022). Highly flexible and compressible polyimide/silica aerogels with integrated double network for thermal insulation and fire-retardancy. J. Mater. Sci. Technol..

[22] Zhang S., Liao Y., Lu K., Wang Z., Wang J., Lai L., Xin W., Xiao Y., Xiong S., Ding F. (2023). Chitosan/silica hybrid aerogels with synergistic capability for superior hydrophobicity and mechanical robustness. Carbohydr. Polym..

[23] Shi T., Jing J., Qian Z., Wu G., Tian G., Liu H., Wang X. (2024). Sandwich-Structured Fluorinated Polyimide Aerogel/Paraffin Phase-Change Composites Simultaneously Enables Gradient Thermal Protection and Electromagnetic Wave Transmission. Adv. Sci..

[24] Williams J.C., Meador M.A.B., McCorkle L.S., Viggiano R.P., Schiraldi D.A. (2020). Thermal shrinkage of polyimide aerogels. ACS Appl. Polym. Mater..

[25] Zhang S., Wang Z., Wang J., Xiao Y., Yang Z., Ji H., Xu G., Xiong S., Li Z., Ding F. (2022). Polyimide aerogels with excellent thermal insulation, hydrophobicity, machinability, and strength evolution at extreme conditions. ACS Appl. Polym. Mater..

[26] Tamaki R., Choi J., Laine R.M. (2003). A polyimide nanocomposite from octa (aminophenyl) silsesquioxane. Chem. Mater..

[27] Unal S., Lin Q., Mourey T.H., Long T.E. (2005). Tailoring the degree of branching: Preparation of poly (ether ester) s via copolymerization of poly (ethylene glycol) oligomers (A_2_) and 1, 3, 5-benzenetricarbonyl trichloride (B_3_). Macromolecules.

[28] Arora N., Flores C., Senarathna M.C., Thompson C.M., Smaldone R.A. (2024). Design, synthesis, and applications of mesoporous covalent organic frameworks. CCS Chem..

[29] Li J., Guo P., Hu C., Pang S., Ma J., Zhao R., Tang S., Cheng H.M. (2022). Fabrication of large aerogel-like carbon/carbon composites with excellent load-bearing capacity and thermal-insulating performance at 1800 °C. ACS Nano.

[30] Li X., Dong G., Liu Z., Zhang X. (2021). Polyimide aerogel fibers with superior flame resistance, strength, hydrophobicity, and flexibility made via a universal sol–gel confined transition strategy. ACS Nano.

[31] Xue T., Zhu C., Yu D., Zhang X., Lai F., Zhang L., Zhang C., Fan W., Liu T. (2023). Fast and scalable production of crosslinked polyimide aerogel fibers for ultrathin thermoregulating clothes. Nat. Commun..

[32] Xie M., Qian G., Ye Q., Zhang Y., Wang M., Deng Z., Yu Y., Chen C., Li H., Li D. (2024). Dual-crosslinked reduced graphene oxide/polyimide aerogels possessing regulable superelasticity, fatigue resistance, and rigidity for thermal insulation and flame retardant protection in harsh conditions. J. Colloid Interface Sci..

[33] Simón-Herrero C., Chen X.Y., Ortiz M.L., Romero A., Valverde J.L., Sánchez-Silva L. (2019). Linear and crosslinked polyimide aerogels: Synthesis and characterization. J. Mater. Res. Technol..

[34] Zhang Z., Pan Y., Gong L., Yao X., Cheng X., Deng Y. (2021). Mechanically strong polyimide aerogels cross-linked with low-cost polymers. RSC Adv..

[35] Shi B., Ma B., Wang C., He H., Qu L., Xu B., Chen Y. (2021). Fabrication and applications of polyimide nano-aerogels. Compos. Part A Appl. Sci. Manuf..

[36] Li M., Wu T., Zhao Z., Li L., Shan T., Wu H., Zboray R., Bernasconi F., Cui Y., Hu P. (2024). Multiscale Manufacturing of Recyclable Polyimide Composite Aerogels. Adv. Mater..

